# Reproductive success in wild and hatchery male coho salmon

**DOI:** 10.1098/rsos.150161

**Published:** 2015-08-12

**Authors:** Bryan D. Neff, Shawn R. Garner, Ian A. Fleming, Mart R. Gross

**Affiliations:** 1 Department of Biology, University of Western Ontario, London, Ontario, Canada N6A 5B7; 2 Department of Ocean Sciences, Memorial University of Newfoundland, St John's, Newfoundland and Labrador, Canada A1C 5S7; 3 Department of Ecology and Evolutionary Biology, University of Toronto, Toronto, Ontario, Canada M5S 3B2

**Keywords:** paternity, hatchery, coho salmon, reproductive success

## Abstract

Salmon produced by hatcheries have lower fitness in the wild than naturally produced salmon, but the factors underlying this difference remain an active area of research. We used genetic parentage analysis of alevins produced by experimentally mixed groups of wild and hatchery coho salmon (*Oncorhynchus kisutch*) to quantify male paternity in spawning hierarchies. We identify factors influencing paternity and revise previously published behavioural estimates of reproductive success for wild and hatchery males. We observed a strong effect of hierarchy size and hierarchy position on paternity: in two-male hierarchies, the first male sired 63% (±29%; s.d.) of the alevins and the second male 37% (±29%); in three-male hierarchies, the first male sired 64% (±26%), the second male 24% (±20%) and the third male 12% (±10%). As previously documented, hatchery males hold inferior positions in spawning hierarchies, but we also discovered that hatchery males had only 55–84% the paternity of wild males when occupying the same position within a spawning hierarchy. This paternity difference may result from inferior performance of hatchery males during sperm competition, female mate choice for wild males, or differential offspring survival. Regardless of its cause, the combination of inferior hierarchical position and inferior success at a position resulted in hatchery males having only half (51%) the reproductive success of wild males.

## 1. Introduction

Salmon are among the world's most economically important fishes, yet many populations are in a state of decline [1]. Hatcheries are often employed to support wild populations but are controversial, because they alter the developmental and selectional environment and consequently the performance of hatchery fish in natural environments [2–5]. A number of studies have documented a decline in the fitness of hatchery fish in the wild ([6–9], but see [10]), but the proximate factors causing the decline remain an active area of research [11,12]. These fitness declines may, in large part, result from differences in breeding behaviour between wild and hatchery fish, and especially the inferiority of hatchery males in spawning competition with wild males [7,13,14]. However, to date no study has directly linked these behavioural deficiencies to *in situ* differences in reproductive success between wild and hatchery males.

Many aspects of salmonid mating systems are well understood, in part because the semelparous life histories of some species mean that reproductive success can be measured in a single breeding season. Female salmon construct and defend nests, whereas male salmon provide no parental care [15,16]. Male reproductive success is limited primarily by access to females and their single egg release within a nest, which leads to intense competition among males for access to nesting females [17]. This pre-spawning competition among males typically results in the formation of a local dominance hierarchy associated with a nesting female, which includes a dominant male nearest the female and may also include one or more subordinate males slightly farther from the female [17,18]. Dominant males subsequently gain an advantage in the position and timing of sperm release [19,20]. Competition for hierarchy positions typically favours males with large body size and elaborate secondary sexual characters such as hooked jaws and dorsal humps [21–23]. The actual distribution of paternity among male salmon at a nest is expected to result from males' competitive success in both pre-spawning competition for positions in dominance hierarchies, and sperm competition, which occurs when sperm from multiple males compete to fertilize an egg. It may also be affected by differences among males in the survival of their offspring [24], or female choice behaviours such as biasing the timing of egg release or directing aggression towards less desirable males [25–27]. Genetic parentage analysis has been used to examine paternity in salmonids and has shown that the alpha male (most dominant individual at the front of the hierarchy, closest to the female) typically achieves the highest paternity (e.g. [20,28–32]). However, the portioning of paternity across males in a hierarchy, and between hatchery and wild males, remains poorly understood.

In this study, we build on one of the earliest experimental programmes to examine the reproductive success of hatchery salmon relative to wild salmon [21,33,34]. In those studies, the reproductive success of hatchery and wild male coho salmon (*Oncorhynchus kisutch*) was estimated from visual observation of individual behaviour over their reproductive lifespan in a spawning channel in British Columbia. Alevins from a subset of the nests were preserved and we now add genetic parentage data to the original behavioural observations with the goal of: (i) quantifying paternity within spawning hierarchies of males; and (ii) re-calculating reproductive success for hatchery and wild males. Together these data provide a rare opportunity for detailed examination of breeding success in wild and hatchery salmon.

## 2. Material and methods

### 2.1 Field site and experimental fish

The fieldwork was conducted from autumn 1988 to spring 1990 in a stream channel replicating the natural spawning grounds of coho salmon in the Oyster River (Vancouver Island, British Columbia, Canada). The channel was constructed in the mid-1980s and used for natural egg incubation. The channel ran parallel to the Oyster River, with an inlet and outlet for the river's flow and river gravel substrate. For our research, the outlet was blocked and salmon were placed in five replicate experimental stream sections at a density similar to the wild (see [33] for full details).

Two non-hatchery enhanced wild populations, Oyster River and Black Creek, were sourced for wild fish. Neither river had any history of hatchery supplementation. Hatchery salmon came from the Quinsam River, which was 22 km north of Oyster River and 25 km north of Black Creek. For 15 years and five generations prior to these experiments, the Quinsam River Hatchery had followed the standard hatchery practice of annual capture of adults entering the hatchery gateway, stripping and crossing gametes, rearing embryos to the smolt stage and releasing smolts into the river. The hatchery fish used in our study were thus bred in the hatchery and released into the river as smolts, whereas the wild fish had spent their whole lives in the wild. Both hatchery and wild salmon were captured in their home river when the adults returned from the ocean to breed. Additional details of the fish used in the spawning channel are given by Fleming & Gross [21,33,34] but in brief, all fish were 3 years of age (no 2 year old precocious ‘jack’ males were used), care was taken to ensure that all fish were treated equally, were at the same stage of ripeness, and that the mean, range and variance in body mass did not differ between wild and hatchery fish (table 1).

**Table 1.** Size of coho salmon in the study. (Data comprise sex, group (all fish in the experimental stream sections or the subset of fish used for parentage analysis), origin, number of individuals (*n*), and mean, range and standard deviation (s.d.) of body mass (kg).)

sex	group (*N*)	origin	*n*	mean	range	s.d.
male	all (100)	wild	50	2.73	1.10–5.24	1.13
		hatchery	50	2.79	1.24–5.58	0.97
	parentage (33)	wild	17	2.88	1.21–5.24	1.25
		hatchery	16	3.00	1.92–5.58	0.99
female	all (96)	wild	48	2.65	1.33–5.05	0.83
		hatchery	48	2.51	1.34–4.55	0.75
	parentage (19)	wild	8	2.66	1.83–3.90	0.67
		hatchery	11	3.05	1.65–4.55	0.84

The research used 100 male and 96 female mature fish, half from the wild and half from the hatchery. All hatchery fish came from the Quinsam River, with four sections of the spawning channel receiving wild fish from the Oyster River (10 wild males, 10 hatchery males, 10 wild females, 10 hatchery females; *n*=40 fish per section) and one section receiving its wild fish from Black Creek (10 wild males, 10 hatchery males, eight wild females, eight hatchery females; *n*=36 fish; fewer females were used in this trial because the availability of Black Creek females was limited). Each adult was externally tagged for individual recognition and its behaviour monitored daily throughout the October–December breeding lifespan. The Oyster River and Black Creek fish displayed similar behavioural patterns [25] and were indistinguishable in all other measures so were combined as ‘wild’ fish in our subsequent analyses.

### 2.2 Behavioural and parentage data

Several times each day, observers on the bank of the stream channel located each individual and recorded a suite of reproductive and non-reproductive behaviours [21,33,34]. For this study, we were interested in: (i) female nest site location and egg release; and (ii) male presence and position in a spawning hierarchy at, or close to the time of egg release. In total, we observed females spawning at 216 nests. The spawning hierarchies recorded at those nests included one to seven males. Males in the spawning hierarchy were scored as first (closest behind female), second or third male (most distal from the female). Few spawning hierarchies had four or more males (29 of 216 hierarchies). The order of male entry into the nest was recorded for observed egg releases, but the distance between male and female at the time of sperm release was not measured.

In March, just prior to fry emergence, the water level in the stream channel was reduced and alevins were collected from a subset of the observed nest sites. Parentage of alevins was then calculated using genetic markers. Tissue samples were taken from the putative mothers (female observed making nest and releasing eggs), putative fathers (the males observed to have spawned at the nest) and from the nest alevins. Alevins are a stage that have yolk sacs and live in the stream gravel before fry emergence: the alevins were cumulatively three to four months in age post-spawning when collected from the experimental channels. A storage failure ruined the 1988 samples, so parentage was genetically examined only from the 1989 breeding. DNA was extracted using a proteinase K digestion followed by phenol–chloroform purification [35]. The putative fathers and mothers at each nest were pre-screened with six microsatellite markers (Ots2, One2, BT73, Ssa14, Omy38 and Omy77) [36–41] to identify an informative subset of the markers (*n*=2–4) that could be used to unambiguously assign paternity to each of the putative fathers. The alevins were then genotyped using only the informative markers for that nest. Paternity within nests was resolved by assigning each alevin to a putative father if it was a genetic match to that male and the putative mother at all markers. This parentage approach takes advantage of the high prior probability that the candidate parents were identified from the behavioural data (i.e. each nest had a reduced pool of candidate parents) [42]. Based on the allele frequencies observed in the putative parents, this approach had an 89.8%±11.3% (mean±s.d.) likelihood of excluding unrelated alevins for each putative parental pair (i.e. there was only a 10.2% chance that an unrelated alevin would genetically match a parental pair by chance). Paternity is reported as the proportion of the alevins in the nest sample that were sired by each male in the spawning hierarchy (the proportions sum to 1). In total, 850 alevins (mean: 40 alevins per nest; range: 10–78), 19 females (wild=8; hatchery=11) and 33 males (wild=17; hatchery=16) were genotyped from 21 nests (individual females spawned in up to two nests, whereas individual males spawned in up to four nests).

### 2.3 Reproductive success

The paternity data from the 21 sampled nests were used to develop an equation for predicting the reproductive success of males in spawning hierarchies. This equation could then be applied to all 100 males in the experiment to estimate their reproductive success from the additional spawning data of Fleming & Gross [21,33,34]. The equation was derived by fitting the paternity data to a linear model with male origin (hatchery, wild) and hierarchy size (1,2,3) as fixed factors, position (1,2,3) as a nested factor within hierarchy size, and body mass as a covariate. There was no significant interaction between male and female origin (*p*=0.83), so female origin was not included in the final paternity model. Lacking paternity data for hierarchies with four or more males, males in fourth or greater positions were for analysis reasons assigned a zero paternity. If we instead used a function of declining paternity by position (for details, see the electronic supplementary material), these males still had negligible paternity. Paternity estimates were multiplied by the number of eggs deposited in each nest (calculated as in [21]) and summed across nests for each male to estimate his total reproductive success. Reproductive success estimates were standardized within stream channel sections by dividing each male's estimate by the total number of eggs available for fertilization in that stream channel section. A linear model was then used to examine the contributions of male origin and body mass to reproductive success. Statistical analyses were performed using JMP (v. 4.0.4, SAS Institute Inc., Cary, NC, USA).

## 3. Results

### 3.1 Behavioural observations

The complete spawning hierarchy participation data for each of the 100 males in the experimental streams are provided in the electronic supplementary material. Statistical analyses of the spawning hierarchies were previously presented in Fleming & Gross [21,33,34]. Briefly, wild males participated in spawning hierarchies at 30% more nests than hatchery males (mean±s.d.: 5.72±3.80 wild versus 4.40±3.12 hatchery) and were the alpha male at 67% more nests than hatchery males (mean±s.d.: 2.70±3.91 hierarchies for wild versus 1.62±2.65 for hatchery). Male body mass was positively correlated with total number of hierarchies (*r*^2^=0.253) and number of hierarchies in the alpha position (*r*^2^=0.397).

### 3.2 Parentage

Male paternities are given in table 2 for the 21 nests that were used for genetic analysis. In total, 776 of the 850 (91%) alevins we genotyped were a genetic match to the nest-tending females, and 729 of those 776 (94%) alevins were a genetic match to one of the males observed in the associated hierarchies. The 74 alevins (=850–776) not matched to the nest-tending females typically matched the genotype of a neighbouring female. The 47 (=776–729) alevins not matched to the males observed in the associated hierarchies may have resulted from unobserved males without a position in the hierarchy, or from males that spawned with the same nest-tending female in a second, adjacent nest. There was no relationship between hierarchy size and the abundance of paternally unmatched alevins (one male hierarchy=3% unmatched alevins (five unmatched of 144 alevins); two males=5% (14 of 255); three males=7% (28 of 388)). The alevins not genetically consistent with both the female and a male observed in the spawning hierarchy were excluded from the analysis, leaving a total alevin sample of 729 ([74+47]/850=14% of alevins rejected).

**Table 2.** Summary of genetic parentage analysis in coho salmon. (Each row represents an individual nest, and includes information on hierarchy size, nest identification (ID), experimental stream section (Exp), female identity, origin (H, hatchery; W, wild), and body mass, male hierarchy composition (identity and origin of male by position), the total number of alevin examined in the genetic analysis, the number of alevin that were excluded because they did not match both the female and a male in the expected hierarchy, the number of alevin assigned to the male in each hierarchy position and the paternity for each male (calculated as the proportion of assigned alevin). The experimental stream sections and male IDs are the same as in the electronic supplementary material 1.)

						male hierarchy						alevin assignment					paternity		
			female			ID			origin					male			male		
size	ID	exp	ID	origin	mass	1	2	3	1	2	3	total	excluded	1	2	3	1	2	3
1	1	3	1	H	2.80	49			H			34	8	26			1.00		
	2	3	2	H	3.80	43			H			45	7	38			1.00		
	3	4	3	W	2.15	72			W			10	0	10			1.00		
	4	4	4	H	2.48	65			H			24	0	24			1.00		
	5	5	5	H	3.42	99			W			43	2	41			1.00		
2	6	3	6	H	2.90	45	47		H	H		45	11	31	3		0.91	0.09	
	7	3	7	W	2.46	56	54		W	W		25	0	21	4		0.84	0.16	
	8	3	8	W	2.89	49	42		H	H		43	1	34	8		0.81	0.19	
	9	5	9	H	2.74	94	86		W	H		45	5	28	12		0.70	0.30	
	10	5	10	W	2.12	97	95		W	W		44	19	16	9		0.64	0.36	
	11	3	1	H	2.80	49	51		H	W		44	14	11	19		0.37	0.63	
	12	5	11	W	2.84	85	91		H	W		37	1	4	32		0.11	0.89	
3	13	3	12	H	3.35	60	50	41	W	H	H	42	0	42	0	0	1.00	0.00	0.00
	14	5	5	H	3.42	99	92	88	W	W	H	43	3	36	2	2	0.90	0.05	0.05
	15	5	13	W	3.12	100	96	87	W	W	H	78	10	57	2	9	0.84	0.03	0.13
	16	4	14	W	1.83	65	66	61	H	H	H	24	2	16	4	2	0.73	0.18	0.09
	17	3	15	H	1.65	47	45	54	H	H	W	44	6	21	17	0	0.55	0.45	0.00
	18	3	16	W	3.90	43	48	52	H	H	W	34	7	16	6	5	0.59	0.22	0.19
	19	5	17	H	4.55	97	92	98	W	W	W	44	12	16	12	4	0.50	0.38	0.13
	20	4	18	H	2.10	73	65	62	W	H	H	58	12	21	13	12	0.46	0.28	0.26
	21	3	19	H	3.80	49	60	53	H	W	W	44	1	7	24	12	0.16	0.56	0.28

**Figure 1.**
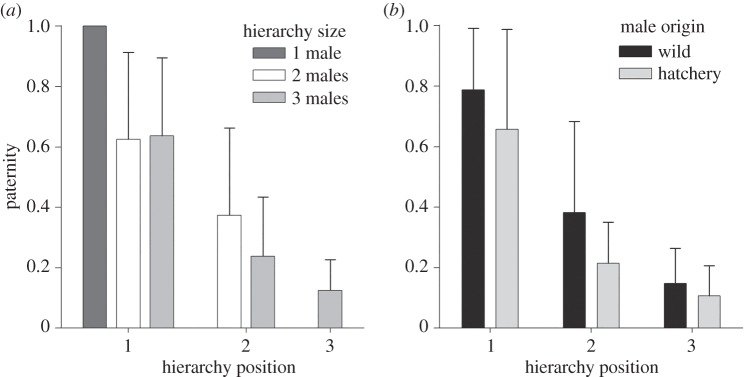
Paternity as a function of position in spawning hierarchies in coho salmon. The data (mean±s.d.) are plotted based on (*a*) total hierarchy size and (*b*) male origin.

The paternity analysis showed that the total size of a hierarchy (*F*_2,38_=23.70, *p*<0.001) and the position of a male within a hierarchy (*F*_3,38_=11.15, *p*<0.001) were the strongest predictors of paternity (figure 1*a* and table 2). In single-male hierarchies, the lone male sired all of the assigned alevins, although the presence of paternally unmatched alevins suggests that unobserved males could have sired up to a maximum of 3% of the alevins in those nests. In two-male hierarchies, the first male sired an average of 63% and the second male 37% of the alevins. In three-male hierarchies, the first male sired an average of 64% of the alevins, the second male 24% and the third male 12%. The addition of a third male thus had no effect on the paternity of the first male. Instead, the third male reduced the paternity of the second male by about 33% (=12%/36%). Across all hierarchy positions, hatchery males had lower paternity than wild males (*F*_1,38_=4.38, *p*=0.043; figure 1*b*). There was no significant effect of body mass on paternity in the model (i.e. once hierarchy size and position were accounted for: *F*_1,38_=2.07, *p*=0.16). Together, the variables included in the model explained 70% of the variance in paternity within male hierarchies.

### 3.3 Reproductive success

**Figure 2.**
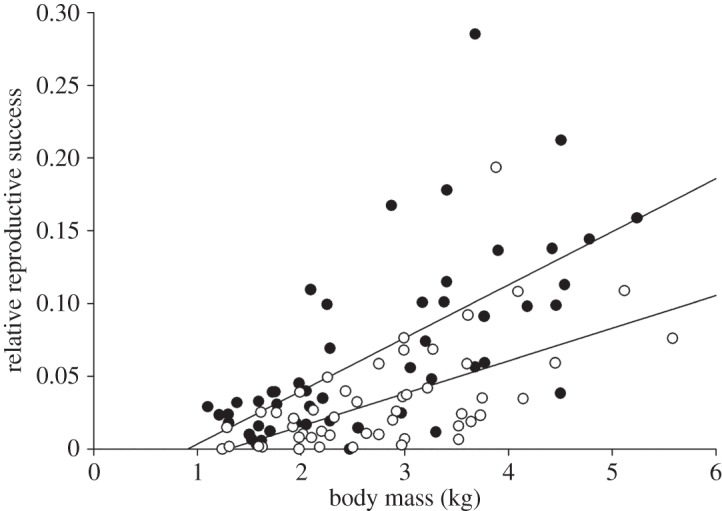
The relationship between male body mass and reproductive success in coho salmon. Reproductive success is expressed as a proportion of the total number of alevins in each experimental stream section. The line of best fit for the data is shown separately for wild males (filled circles) and hatchery males (open circles). There was no significant difference in the slopes of these lines.

Reproductive success for all 100 males was estimated using the spawning hierarchy observations of Fleming & Gross [21,33,34] and the equation derived here from the paternity analysis (see the electronic supplementary material for details and estimates for each male). This analysis revealed a significant positive relationship between reproductive success and body mass (*F*_1,96_=61.58, *p*<0.001; figure 2). There was a significant effect of male origin on reproductive success (*F*_1,96_=20.36, *p*<0.001), with hatchery males having only 51% of the reproductive success of wild males (mean proportion of alevins sired per male (±s.d.): 0.0336±0.0363 for hatchery males versus 0.0664±0.0615 for wild males). There was no significant interaction between male origin and body mass (*F*_1,96_=3.51, *p*=0.064).

## 4. Discussion

In salmonids, males typically compete for access to spawning females and form dominance hierarchies that affect the timing and position of sperm release. Previous studies have shown that alpha positions in spawning hierarchies are associated with higher paternity through an advantage in sperm competition [19,20,31,32], and that the intense competition among males for hierarchy positions favours elaborate secondary sexual characters and large body size [21–23]. Examining paternity in coho salmon nests, we similarly found that alpha males had high paternity. This paternity advantage was especially pronounced when males were able to exclude all other rivals from a spawning hierarchy, as in the absence of an observed competitor the first male achieved at least 97% paternity. When a second male was present in a hierarchy, the first male fertilized 63% of the eggs, with no additional reduction in paternity when a third male was present. Instead, the third male's paternity came from the second male's share. This distribution of paternity could explain the behavioural observation that the first male in a hierarchy typically fights with the second male and ignores the third, whereas the second male fights with both the first and third males [21,33,34]. Within hierarchies, male body mass did not have a significant effect on paternity when controlling for position. However, large males obtained dominant hierarchy positions more frequently than small males [33], and this positional advantage led to a strong positive relationship between male body mass and reproductive success.

Wild and hatchery males are known to differ in their ability to obtain preferred positions in spawning hierarchies (e.g. [7,13,14]), but the breeding success of these males may also differ as a result of additional processes that include sperm competition, differential survival and female choice. Indeed, these processes are implicated in our study, in which hatchery salmon had lower paternity than wild salmon when both held equivalent positions in spawning hierarchies. This paternity difference could arise because hatchery males produce inferior sperm, which have lower velocity or motility than the sperm of wild males. However, several studies have found that hatchery and wild salmonids do not consistently differ in sperm motility or velocity [43–45], which suggests that sperm performance is unlikely to be the primary cause of the observed differences in paternity. Instead, hatchery fish might have low paternity because they are slower than wild males to release sperm or are more likely to be excluded from preferred spawning positions immediately adjacent to females, as both characteristics have been linked to low success in sperm competition [19,31,46,47]. Alternatively, the paternity difference could arise because the offspring of hatchery males have lower survival than the offspring of wild males during the three to four month post-spawn period (eggs and alevins), as was observed in a previous study of steelhead trout (*Oncorhynchus mykiss*) [48]. We observed no significant interaction between male and female origin on paternity, which suggests that genetic incompatibilities between wild and hatchery fish are unlikely to explain the paternity difference. Finally, the paternity difference might also have been caused by a female behavioural bias—a form of cryptic female choice—for wild males over hatchery males. A previous study on coho salmon, for example, showed that females directed greater aggression towards hatchery males than wild males [49]. Regardless of the specific mechanism, our paternity data clearly indicate that processes other than competition for spawning hierarchy positions are contributing to the inferior breeding performance of hatchery males relative to wild males.

It has been widely reported that hatchery fish produce fewer progeny than wild fish in natural habitats [6–8]. In one review, it was estimated that each generation of captive rearing is associated with about a 40% decline in lifetime reproductive success [6]. This difference can be traced in large part to less-effective reproductive behaviours, including the low success of hatchery males during pre-spawning competition for mating opportunities [7,13,50]. For example, based on behavioural observations of coho salmon, Fleming & Gross [33] estimated that hatchery males fertilized 62% as many eggs as wild males. Combining these previous behavioural observations with the genetic paternity data reported here, we were able to re-calculate the reproductive success of these coho salmon. We confirmed the general conclusions of this earlier study, but the lower paternity of hatchery males across all hierarchy positions meant that we now estimate that hatchery males actually sired only 51% as many alevins as wild males. By contrast, the hatchery females in the experimental streams produced an estimated 82% as many alevins as wild females [33]. Differential success during breeding thus makes a substantial contribution to the lower lifetime reproductive success of hatchery fish, with especially pronounced effects for males.

Differences in breeding success between wild and hatchery fish could have important management consequences, because they suggest that hatchery fish will have lower fitness than wild fish in mixed populations. This effect could in part explain why a number of studies have failed to link supplementation hatcheries to a clear improvement in fish abundance [51,52]. Our data add to the growing evidence that hatchery-produced fish have reduced breeding success in mixed populations and are the first to implicate processes other than competition for spawning hierarchy positions as part of the deficiency. Hatchery programmes may aid conservation of endangered fishes in a few situations [53–55], but the additional findings here of breeding inferiority further the important concern about using hatchery fish to supplement wild populations [6,11,12,56].

## Supplementary Material

Spawning data and functions used to estimate paternity in coho salmon.

## Data accessibility

The datasets supporting this article have been uploaded as part of the electronic supplementary material.
