# Supplementary material for: Reproductive success in wild and hatchery male coho salmon
Source: R Soc Open Sci. 2015 Aug 12;2(8):150161. doi: 10.1098/rsos.150161 (PMC4555853; doi:10.1098/rsos.150161)
Supplement: Spawning data and functions used to estimate paternity in coho salmon. [file rsos150161supp1.doc]

Supplementary material

*Functions used to estimate paternity in coho salmon*

Equation [1] was derived by fitting the genetic paternity data to a linear model that included body mass, male type (hatchery or wild), and combined hierarchy size and position (success in a position varied with hierarchy size). This function explained 70% of the variation in paternity among males and was used for all calculations of reproductive success presented in the main text of the article. No paternity was assigned to males in position 4 or higher using this function.

[1] Paternity = 0.7076 + Hierarchy size (position) – 0.0488 *M* + 0.1292 *T*

for Hierarchy size (position) 1 (1) = 0.4132

2 (1) = 0.0395

2 (2) = -0.2979

3 (1) = 0.0411

3 (2) = -0.3620

3 (3) = -0.5310

Where *M* is body mass (kg) and *T* is male type (Hatchery=0, Wild=1)

We also used our genetic data to derive an equation that could assign paternity to males in all hierarchy positions. First, we used a continuous function to capture the effects of hierarchy size and position on paternity. Our genetic data suggested that each male in a hierarchy reduceed the paternity of the male ahead of him by 37% (similar to the 50% rule previously used by Fleming and Gross), but did not affect the paternity of males more than one position ahead of him. For example, in a three male hierarchy the first male gains 63% paternity (= 100% - [100%× 0.37]), the second male receives 23% paternity (= 37% - [37%× 0.37]) and the third male receives 14% paternity (= [37%× 0.37]). This relationship is expressed in equation [2].

[2] expected paternity = 0.37^A^ × 0.63^B^

Where *A* is the number of males ahead of the focal individual in the spawning hierarchy and *B* is the presence of another male behind the focal male in the hierarchy (Yes=1, No=0).

Equation [3] was then derived analogously to equation [1] by fitting the genetic paternity data to a linear model that included body mass, male type (hatchery or wild), and the expected paternity calculated using equation [2]. This function explained 69% of the variation in parentage among males and produced reproductive success estimates that were similar to equation [1] (supplementary table).

[3] Paternity = 0.0198 + 1.0657 (0.37^A^ × 0.63^B^) – 0.0358 *M* + 0.1286 *T*

*Supplementary table*

Summary of visual spawning observations and reproductive success for each male coho salmon. Each row represents an individual male, and includes information on year, experimental stream section (Exp), origin, individual ID, body mass (kg), number of spawning hierarchies by position, and estimated reproductive success expressed as total number of eggs and as the proportion of eggs within each experiment. Reproductive success was calculated by assigning paternity to only the first 3 males (equation [1]) and all males in the spawning hierarchy (equation [3]).

|  |  |  |  |  |  | Hierarchy size and position | | | | | | | | | | | | |  | Lifetime reproductive success | | | | |
| --- | --- | --- | --- | --- | --- | --- | --- | --- | --- | --- | --- | --- | --- | --- | --- | --- | --- | --- | --- | --- | --- | --- | --- | --- |
|  |  |  |  |  |  | **1** |  | **2** | |  | **3** | | |  | **4+** | | | |  | First 3 | |  | All | |
| Year | Exp | Origin | ID | Mass |  | 1 |  | 1 | 2 |  | 1 | 2 | 3 |  | 1 | 2 | 3 | 4+ |  | # | % |  | # | % |
| 1988 | 1 | Hatchery | 1 | 3.27 |  | 0 |  | 2 | 1 |  | 1 | 6 | 1 |  | 0 | 1 | 0 | 0 |  | 3171 | 0.068 |  | 3193 | 0.068 |
|  |  |  | 2 | 1.63 |  | 0 |  | 0 | 0 |  | 0 | 0 | 1 |  | 0 | 0 | 0 | 0 |  | 64 | 0.001 |  | 64 | 0.001 |
|  |  |  | 3 | 1.28 |  | 0 |  | 0 | 1 |  | 0 | 1 | 0 |  | 0 | 0 | 1 | 1 |  | 634 | 0.014 |  | 692 | 0.015 |
|  |  |  | 4 | 1.24 |  | 0 |  | 0 | 0 |  | 0 | 0 | 0 |  | 0 | 0 | 0 | 0 |  | 0 | 0.000 |  | 0 | 0.000 |
|  |  |  | 5 | 1.61 |  | 0 |  | 0 | 1 |  | 0 | 1 | 2 |  | 0 | 0 | 0 | 2 |  | 1198 | 0.026 |  | 1175 | 0.025 |
|  |  |  | 6 | 4.09 |  | 0 |  | 2 | 1 |  | 3 | 1 | 0 |  | 2 | 0 | 1 | 0 |  | 4946 | 0.106 |  | 5051 | 0.108 |
|  |  |  | 7 | 3.02 |  | 0 |  | 2 | 0 |  | 0 | 1 | 0 |  | 1 | 0 | 1 | 0 |  | 1752 | 0.038 |  | 1737 | 0.037 |
|  |  |  | 8 | 1.59 |  | 0 |  | 0 | 0 |  | 0 | 0 | 1 |  | 0 | 0 | 0 | 0 |  | 83 | 0.002 |  | 83 | 0.002 |
|  |  |  | 9 | 3.75 |  | 0 |  | 1 | 1 |  | 1 | 1 | 0 |  | 0 | 0 | 0 | 0 |  | 1635 | 0.035 |  | 1635 | 0.035 |
|  |  |  | 10 | 2.97 |  | 0 |  | 0 | 1 |  | 0 | 0 | 0 |  | 0 | 0 | 0 | 1 |  | 121 | 0.003 |  | 121 | 0.003 |
|  |  | Wild | 11 | 3.41 |  | 1 |  | 0 | 0 |  | 3 | 1 | 1 |  | 0 | 2 | 0 | 0 |  | 5225 | 0.112 |  | 5364 | 0.115 |
|  |  |  | 12 | 4.51 |  | 1 |  | 2 | 0 |  | 7 | 0 | 0 |  | 3 | 0 | 0 | 0 |  | 9953 | 0.213 |  | 9909 | 0.212 |
|  |  |  | 13 | 1.38 |  | 0 |  | 0 | 1 |  | 0 | 0 | 2 |  | 0 | 0 | 0 | 0 |  | 1484 | 0.032 |  | 1484 | 0.032 |
|  |  |  | 14 | 2.87 |  | 1 |  | 1 | 0 |  | 3 | 4 | 2 |  | 0 | 1 | 1 | 0 |  | 7700 | 0.165 |  | 7814 | 0.167 |
|  |  |  | 15 | 3.26 |  | 0 |  | 1 | 0 |  | 0 | 0 | 1 |  | 0 | 1 | 1 | 0 |  | 2191 | 0.047 |  | 2247 | 0.048 |
|  |  |  | 16 | 3.30 |  | 0 |  | 0 | 0 |  | 0 | 1 | 0 |  | 0 | 0 | 1 | 1 |  | 642 | 0.014 |  | 546 | 0.012 |
|  |  |  | 17 | 1.10 |  | 0 |  | 0 | 2 |  | 0 | 0 | 2 |  | 0 | 0 | 0 | 0 |  | 1351 | 0.029 |  | 1351 | 0.029 |
|  |  |  | 18 | 3.06 |  | 0 |  | 1 | 1 |  | 0 | 1 | 2 |  | 0 | 1 | 0 | 1 |  | 2624 | 0.056 |  | 2607 | 0.056 |
|  |  |  | 19 | 1.62 |  | 0 |  | 0 | 1 |  | 0 | 0 | 0 |  | 0 | 0 | 0 | 0 |  | 270 | 0.006 |  | 270 | 0.006 |
|  |  |  | 20 | 2.09 |  | 0 |  | 0 | 1 |  | 0 | 0 | 3 |  | 0 | 0 | 0 | 1 |  | 1654 | 0.035 |  | 1354 | 0.029 |
|  | 2 | Hatchery | 21 | 3.61 |  | 2 |  | 1 | 0 |  | 1 | 0 | 0 |  | 0 | 1 | 0 | 0 |  | 2954 | 0.091 |  | 2974 | 0.092 |
|  |  |  | 22 | 1.76 |  | 0 |  | 0 | 1 |  | 0 | 0 | 1 |  | 0 | 1 | 0 | 1 |  | 756 | 0.023 |  | 810 | 0.025 |
|  |  |  | 23 | 2.99 |  | 1 |  | 2 | 2 |  | 1 | 0 | 0 |  | 0 | 1 | 0 | 0 |  | 2170 | 0.067 |  | 2194 | 0.068 |
|  |  |  | 24 | 2.88 |  | 0 |  | 0 | 0 |  | 1 | 1 | 0 |  | 0 | 2 | 0 | 0 |  | 591 | 0.018 |  | 641 | 0.020 |
|  |  |  | 25 | 1.31 |  | 0 |  | 0 | 0 |  | 0 | 0 | 1 |  | 0 | 0 | 0 | 0 |  | 51 | 0.002 |  | 51 | 0.002 |
|  |  |  | 26 | 2.43 |  | 1 |  | 0 | 1 |  | 0 | 0 | 1 |  | 0 | 0 | 0 | 0 |  | 1283 | 0.040 |  | 1283 | 0.040 |
|  |  |  | 27 | 2.26 |  | 0 |  | 2 | 1 |  | 0 | 1 | 0 |  | 0 | 0 | 0 | 0 |  | 1595 | 0.049 |  | 1595 | 0.049 |
|  |  |  | 28 | 2.18 |  | 0 |  | 0 | 0 |  | 0 | 0 | 1 |  | 0 | 0 | 0 | 1 |  | 38 | 0.001 |  | 38 | 0.001 |
|  |  |  | 29 | 2.10 |  | 0 |  | 0 | 1 |  | 0 | 0 | 0 |  | 0 | 0 | 0 | 2 |  | 255 | 0.008 |  | 255 | 0.008 |
|  |  |  | 30 | 2.99 |  | 0 |  | 2 | 0 |  | 0 | 0 | 0 |  | 1 | 0 | 1 | 0 |  | 2530 | 0.078 |  | 2471 | 0.076 |
|  |  | Wild | 31 | 1.72 |  | 0 |  | 0 | 3 |  | 0 | 1 | 1 |  | 0 | 0 | 1 | 0 |  | 1246 | 0.038 |  | 1267 | 0.039 |
|  |  |  | 32 | 1.77 |  | 0 |  | 0 | 1 |  | 0 | 0 | 1 |  | 1 | 0 | 0 | 2 |  | 1111 | 0.034 |  | 992 | 0.031 |
|  |  |  | 33 | 1.50 |  | 0 |  | 0 | 0 |  | 0 | 0 | 1 |  | 0 | 0 | 0 | 0 |  | 323 | 0.010 |  | 323 | 0.010 |
|  |  |  | 34 | 3.77 |  | 0 |  | 2 | 0 |  | 1 | 1 | 0 |  | 1 | 0 | 0 | 0 |  | 2935 | 0.091 |  | 2951 | 0.091 |
|  |  |  | 35 | 3.41 |  | 1 |  | 3 | 1 |  | 2 | 0 | 1 |  | 1 | 0 | 1 | 0 |  | 5779 | 0.179 |  | 5758 | 0.178 |
|  |  |  | 36 | 1.70 |  | 0 |  | 0 | 0 |  | 0 | 1 | 0 |  | 0 | 1 | 0 | 0 |  | 367 | 0.011 |  | 396 | 0.012 |
|  |  |  | 37 | 2.09 |  | 2 |  | 0 | 2 |  | 0 | 2 | 0 |  | 0 | 0 | 2 | 0 |  | 3488 | 0.108 |  | 3540 | 0.109 |
|  |  |  | 38 | 2.97 |  | 0 |  | 1 | 0 |  | 0 | 1 | 0 |  | 0 | 0 | 1 | 0 |  | 782 | 0.024 |  | 797 | 0.025 |
|  |  |  | 39 | 3.17 |  | 0 |  | 2 | 0 |  | 2 | 0 | 0 |  | 2 | 0 | 0 | 0 |  | 3273 | 0.101 |  | 3260 | 0.101 |
|  |  |  | 40 | 1.30 |  | 0 |  | 0 | 2 |  | 0 | 0 | 0 |  | 0 | 0 | 0 | 1 |  | 841 | 0.026 |  | 770 | 0.024 |
| 1989 | 3 | Hatchery | 41 | 2.20 |  | 0 |  | 1 | 0 |  | 0 | 0 | 2 |  | 0 | 0 | 0 | 0 |  | 606 | 0.012 |  | 606 | 0.012 |
|  |  |  | 42 | 1.92 |  | 0 |  | 0 | 2 |  | 0 | 0 | 0 |  | 0 | 0 | 0 | 2 |  | 762 | 0.015 |  | 762 | 0.015 |
|  |  |  | 43 | 2.75 |  | 1 |  | 1 | 1 |  | 1 | 0 | 0 |  | 0 | 0 | 1 | 0 |  | 2902 | 0.058 |  | 2924 | 0.058 |
|  |  |  | 44 | 2.63 |  | 0 |  | 0 | 1 |  | 0 | 0 | 0 |  | 0 | 0 | 0 | 0 |  | 528 | 0.011 |  | 528 | 0.011 |
|  |  |  | 45 | 2.54 |  | 0 |  | 1 | 1 |  | 0 | 1 | 1 |  | 0 | 1 | 0 | 1 |  | 1546 | 0.031 |  | 1611 | 0.032 |
|  |  |  | 46 | 2.02 |  | 0 |  | 0 | 1 |  | 0 | 0 | 0 |  | 0 | 1 | 0 | 1 |  | 470 | 0.009 |  | 508 | 0.010 |
|  |  |  | 47 | 1.99 |  | 0 |  | 0 | 2 |  | 1 | 1 | 1 |  | 0 | 1 | 1 | 0 |  | 1894 | 0.038 |  | 1943 | 0.039 |
|  |  |  | 48 | 2.32 |  | 0 |  | 0 | 2 |  | 0 | 1 | 0 |  | 0 | 0 | 0 | 2 |  | 1082 | 0.022 |  | 1082 | 0.022 |
|  |  |  | 49 | 3.88 |  | 3 |  | 7 | 0 |  | 5 | 0 | 0 |  | 0 | 1 | 0 | 0 |  | 9665 | 0.193 |  | 9677 | 0.193 |
|  |  |  | 50 | 2.92 |  | 0 |  | 0 | 2 |  | 0 | 3 | 0 |  | 0 | 0 | 2 | 1 |  | 1241 | 0.025 |  | 1292 | 0.026 |
|  |  | Wild | 51 | 2.25 |  | 2 |  | 1 | 4 |  | 0 | 3 | 0 |  | 0 | 0 | 0 | 0 |  | 4967 | 0.099 |  | 4967 | 0.099 |
|  |  |  | 52 | 2.28 |  | 0 |  | 0 | 1 |  | 0 | 0 | 1 |  | 0 | 0 | 1 | 0 |  | 955 | 0.019 |  | 963 | 0.019 |
|  |  |  | 53 | 1.59 |  | 0 |  | 0 | 1 |  | 0 | 0 | 1 |  | 0 | 0 | 0 | 0 |  | 789 | 0.016 |  | 789 | 0.016 |
|  |  |  | 54 | 1.59 |  | 0 |  | 1 | 1 |  | 0 | 0 | 2 |  | 0 | 0 | 1 | 1 |  | 1746 | 0.035 |  | 1630 | 0.033 |
|  |  |  | 55 | 1.75 |  | 0 |  | 0 | 2 |  | 0 | 0 | 2 |  | 0 | 1 | 0 | 0 |  | 1924 | 0.038 |  | 1963 | 0.039 |
|  |  |  | 56 | 2.28 |  | 1 |  | 2 | 1 |  | 0 | 0 | 0 |  | 0 | 0 | 0 | 0 |  | 3459 | 0.069 |  | 3459 | 0.069 |
|  |  |  | 57 | 1.53 |  | 0 |  | 0 | 1 |  | 0 | 0 | 0 |  | 0 | 0 | 0 | 0 |  | 329 | 0.007 |  | 329 | 0.007 |
|  |  |  | 58 | 2.47 |  | 0 |  | 0 | 0 |  | 0 | 0 | 0 |  | 0 | 0 | 0 | 0 |  | 0 | 0.000 |  | 0 | 0.000 |
|  |  |  | 59 | 2.55 |  | 1 |  | 0 | 0 |  | 0 | 0 | 0 |  | 0 | 1 | 0 | 2 |  | 862 | 0.017 |  | 723 | 0.014 |
|  |  |  | 60 | 3.68 |  | 2 |  | 9 | 0 |  | 3 | 1 | 0 |  | 6 | 0 | 0 | 0 |  | 14287 | 0.286 |  | 14261 | 0.285 |
|  | 4 | Hatchery | 61 | 2.75 |  | 0 |  | 0 | 0 |  | 0 | 1 | 2 |  | 0 | 0 | 2 | 1 |  | 359 | 0.008 |  | 431 | 0.010 |
|  |  |  | 62 | 3.52 |  | 0 |  | 0 | 0 |  | 0 | 1 | 1 |  | 0 | 1 | 0 | 1 |  | 237 | 0.006 |  | 283 | 0.007 |
|  |  |  | 63 | 3.22 |  | 1 |  | 0 | 0 |  | 1 | 0 | 1 |  | 0 | 0 | 1 | 0 |  | 1776 | 0.041 |  | 1802 | 0.042 |
|  |  |  | 64 | 2.28 |  | 0 |  | 0 | 1 |  | 0 | 0 | 0 |  | 0 | 0 | 1 | 0 |  | 353 | 0.008 |  | 403 | 0.009 |
|  |  |  | 65 | 4.45 |  | 1 |  | 0 | 1 |  | 2 | 1 | 0 |  | 0 | 0 | 0 | 0 |  | 2545 | 0.059 |  | 2545 | 0.059 |
|  |  |  | 66 | 3.60 |  | 2 |  | 2 | 1 |  | 0 | 2 | 0 |  | 0 | 1 | 0 | 0 |  | 2439 | 0.057 |  | 2521 | 0.059 |
|  |  |  | 67 | 5.12 |  | 1 |  | 1 | 2 |  | 1 | 0 | 0 |  | 4 | 0 | 1 | 0 |  | 4420 | 0.103 |  | 4683 | 0.109 |
|  |  |  | 68 | 3.52 |  | 0 |  | 2 | 0 |  | 0 | 0 | 0 |  | 0 | 0 | 0 | 0 |  | 680 | 0.016 |  | 680 | 0.016 |
|  |  |  | 69 | 1.98 |  | 0 |  | 0 | 0 |  | 0 | 0 | 0 |  | 0 | 1 | 0 | 0 |  | 256 | 0.006 |  | 341 | 0.008 |
|  |  |  | 70 | 3.64 |  | 0 |  | 0 | 2 |  | 0 | 1 | 1 |  | 0 | 1 | 0 | 1 |  | 768 | 0.018 |  | 807 | 0.019 |
|  |  | Wild | 71 | 4.50 |  | 1 |  | 0 | 1 |  | 1 | 0 | 0 |  | 0 | 0 | 0 | 1 |  | 1661 | 0.039 |  | 1646 | 0.038 |
|  |  |  | 72 | 3.38 |  | 2 |  | 1 | 0 |  | 0 | 0 | 1 |  | 1 | 0 | 0 | 1 |  | 4323 | 0.100 |  | 4348 | 0.101 |
|  |  |  | 73 | 4.78 |  | 0 |  | 6 | 0 |  | 2 | 0 | 0 |  | 1 | 0 | 0 | 0 |  | 6224 | 0.144 |  | 6210 | 0.144 |
|  |  |  | 74 | 1.30 |  | 0 |  | 0 | 0 |  | 0 | 1 | 1 |  | 0 | 0 | 0 | 2 |  | 1090 | 0.025 |  | 774 | 0.018 |
|  |  |  | 75 | 2.05 |  | 1 |  | 0 | 4 |  | 0 | 0 | 0 |  | 0 | 0 | 0 | 2 |  | 1888 | 0.044 |  | 1714 | 0.040 |
|  |  |  | 76 | 4.54 |  | 3 |  | 1 | 0 |  | 1 | 0 | 0 |  | 0 | 0 | 0 | 0 |  | 4862 | 0.113 |  | 4862 | 0.113 |
|  |  |  | 77 | 2.21 |  | 0 |  | 1 | 1 |  | 1 | 0 | 0 |  | 0 | 0 | 1 | 1 |  | 1544 | 0.036 |  | 1497 | 0.035 |
|  |  |  | 78 | 4.46 |  | 3 |  | 2 | 0 |  | 0 | 1 | 0 |  | 0 | 1 | 0 | 0 |  | 4186 | 0.097 |  | 4252 | 0.099 |
|  |  |  | 79 | 2.05 |  | 0 |  | 0 | 0 |  | 0 | 0 | 2 |  | 0 | 1 | 0 | 1 |  | 828 | 0.019 |  | 725 | 0.017 |
|  |  |  | 80 | 3.77 |  | 1 |  | 0 | 3 |  | 0 | 1 | 0 |  | 0 | 0 | 0 | 0 |  | 2553 | 0.059 |  | 2553 | 0.059 |
|  | 5 | Hatchery | 81 | 3.73 |  | 0 |  | 0 | 1 |  | 1 | 0 | 0 |  | 0 | 0 | 0 | 0 |  | 1037 | 0.023 |  | 1037 | 0.023 |
|  |  |  | 82 | 4.14 |  | 2 |  | 1 | 0 |  | 0 | 0 | 0 |  | 0 | 0 | 0 | 0 |  | 1557 | 0.034 |  | 1557 | 0.034 |
|  |  |  | 83 | 3.55 |  | 1 |  | 0 | 0 |  | 0 | 0 | 0 |  | 0 | 0 | 1 | 0 |  | 1084 | 0.024 |  | 1089 | 0.024 |
|  |  |  | 84 | 1.93 |  | 0 |  | 0 | 2 |  | 0 | 0 | 2 |  | 0 | 0 | 2 | 0 |  | 832 | 0.018 |  | 944 | 0.021 |
|  |  |  | 85 | 5.58 |  | 0 |  | 2 | 1 |  | 1 | 1 | 0 |  | 2 | 0 | 0 | 0 |  | 3270 | 0.072 |  | 3429 | 0.076 |
|  |  |  | 86 | 3.00 |  | 0 |  | 0 | 1 |  | 0 | 0 | 0 |  | 0 | 0 | 1 | 0 |  | 281 | 0.006 |  | 314 | 0.007 |
|  |  |  | 87 | 2.12 |  | 0 |  | 0 | 0 |  | 0 | 1 | 1 |  | 1 | 0 | 1 | 0 |  | 1191 | 0.026 |  | 1203 | 0.027 |
|  |  |  | 88 | 2.50 |  | 0 |  | 0 | 0 |  | 0 | 0 | 1 |  | 0 | 0 | 0 | 0 |  | 50 | 0.001 |  | 50 | 0.001 |
|  |  |  | 89 | 2.98 |  | 1 |  | 0 | 1 |  | 0 | 0 | 1 |  | 0 | 1 | 0 | 1 |  | 1552 | 0.034 |  | 1615 | 0.036 |
|  |  |  | 90 | 1.98 |  | 0 |  | 0 | 0 |  | 0 | 0 | 0 |  | 0 | 0 | 0 | 0 |  | 0 | 0.000 |  | 0 | 0.000 |
|  |  | Wild | 91 | 1.21 |  | 0 |  | 0 | 1 |  | 0 | 0 | 0 |  | 0 | 1 | 0 | 2 |  | 1440 | 0.032 |  | 1055 | 0.023 |
|  |  |  | 92 | 4.42 |  | 4 |  | 1 | 1 |  | 0 | 5 | 1 |  | 0 | 0 | 0 | 0 |  | 6221 | 0.138 |  | 6221 | 0.138 |
|  |  |  | 93 | 4.18 |  | 2 |  | 2 | 1 |  | 0 | 1 | 0 |  | 0 | 2 | 0 | 0 |  | 4243 | 0.094 |  | 4426 | 0.098 |
|  |  |  | 94 | 1.95 |  | 0 |  | 1 | 0 |  | 0 | 0 | 0 |  | 0 | 0 | 0 | 1 |  | 1018 | 0.023 |  | 814 | 0.018 |
|  |  |  | 95 | 3.68 |  | 2 |  | 0 | 2 |  | 0 | 0 | 2 |  | 0 | 0 | 0 | 0 |  | 2546 | 0.056 |  | 2546 | 0.056 |
|  |  |  | 96 | 1.98 |  | 0 |  | 0 | 1 |  | 0 | 2 | 1 |  | 0 | 1 | 0 | 0 |  | 1940 | 0.043 |  | 2038 | 0.045 |
|  |  |  | 97 | 5.24 |  | 1 |  | 3 | 0 |  | 5 | 0 | 0 |  | 1 | 0 | 0 | 1 |  | 7126 | 0.158 |  | 7168 | 0.159 |
|  |  |  | 98 | 1.57 |  | 0 |  | 0 | 0 |  | 0 | 0 | 1 |  | 0 | 0 | 0 | 1 |  | 323 | 0.007 |  | 141 | 0.003 |
|  |  |  | 99 | 3.20 |  | 1 |  | 1 | 2 |  | 1 | 0 | 0 |  | 0 | 0 | 0 | 0 |  | 3337 | 0.074 |  | 3337 | 0.074 |
|  |  |  | 100 | 3.90 |  | 1 |  | 4 | 1 |  | 2 | 0 | 0 |  | 1 | 0 | 0 | 0 |  | 6096 | 0.135 |  | 6158 | 0.136 |
